# Supplementary material for: An Observational Real-Life Study with a New Infant Formula in Infants with Functional Gastro-Intestinal Disorders
Source: Nutrients. 2021 Sep 23;13(10):3336. doi: 10.3390/nu13103336 (PMC8539302; doi:10.3390/nu13103336)
Supplement: Supplementary file 1 [file nutrients-13-03336-s001.zip › nutrients-1370336-supplementary.pdf]

nutrients

MDPI

Supplementary Materials

Table S1. CoMiSS score—Multivariate analysis.

Covariates tested		Estimate	SE	95% CI	p-value
**CoMiSS at baseline**		0.09	0.01	0.07 ; 0.1	<.0001
**Age at baseline**	Months	0.02	0.04	-0.06 ; 0.11	0.5612
**Sex**	Male	-0.05	0.04	-0.13 ; 0.03	0.2448
**Weight at baseline**	Kg	-0.03	0.04	-0.11 ; 0.05	0.4752
**BMI at birth**	Kg/m^2	0.01	0.02	-0.02 ; 0.05	0.4468
**Gestational age**	Weeks	0.00	0.01	-0.02 ; 0.03	0.7324
**Mode of delivery**	C-section	0.12	0.05	0.03 ; 0.21	0.012
**Presence of adverse event**	Yes	0.02	0.04	-0.05 ; 0.1	0.577
**Time**	Day	-0.01	0.00	-0.02 ; 0	0.0054

SE: Standard error; C-section: Cesarean delivery; CI: Confidence interval
Mixed effect model with covariates as fixed effect and patient as random effect. Covariance of random effect unstructured
Estimated using proc glimmix with gamma distribution and log link (CoMiSS score is skewed and looks like count data, see Figure 1)

Table S2. QUALIN score—Multivariate analysis.

Covariates tested		Estimate	SE	95% CI	p-value
**QUALIN at baseline**		0.69	0.03	0.63 ; 0.76	<.0001
**Age at baseline**	Months	1.66	0.49	0.7 ; 2.63	0.0008
**Weight at baseline**	Kg	-0.95	0.46	-1.86 ; -0.04	0.0413
**Gestational age**	Weeks	-0.29	0.19	-0.66 ; 0.09	0.1324
**Crying (reference=No)**	Yes	1.18	0.74	-0.29 ; 2.64	0.1146
**Time**	Day	0.22	0.04	0.15 ; 0.3	<.0001

SE: Standard error; CI: Confidence interval
Mixed effect model with each covariate as fixed effect and patient as random effect. Covariance of random effect unstructured
Estimated using proc glimmix with normal distribution

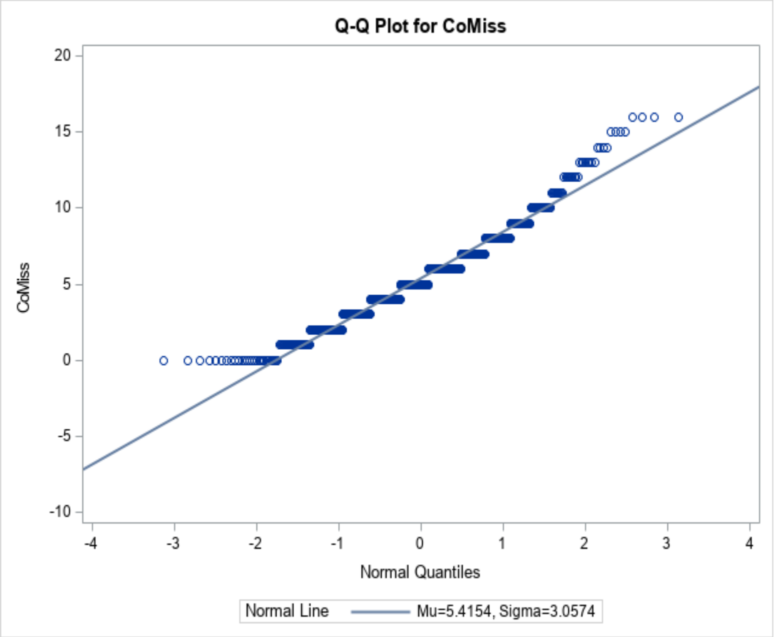

Figure S1. Q-Q Plot for CoMiSS.

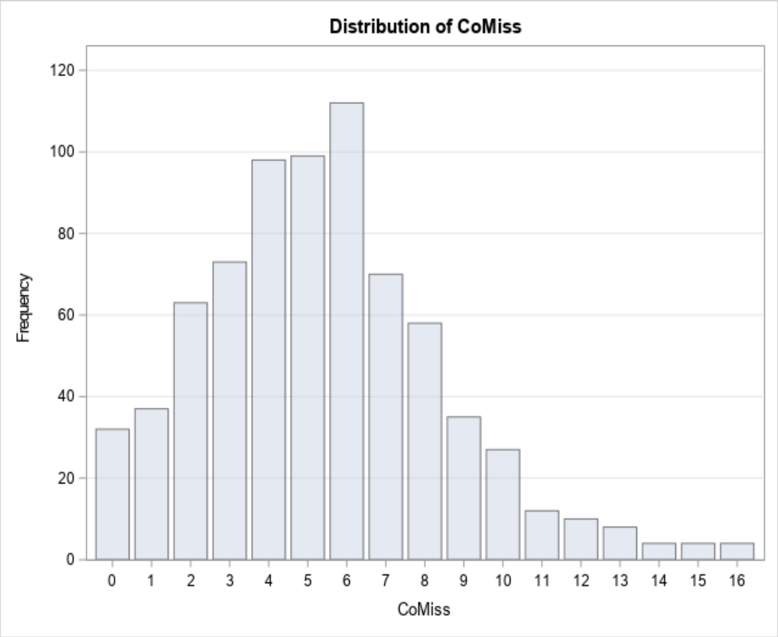

Figure S2. Distribution of CoMiSS scores.

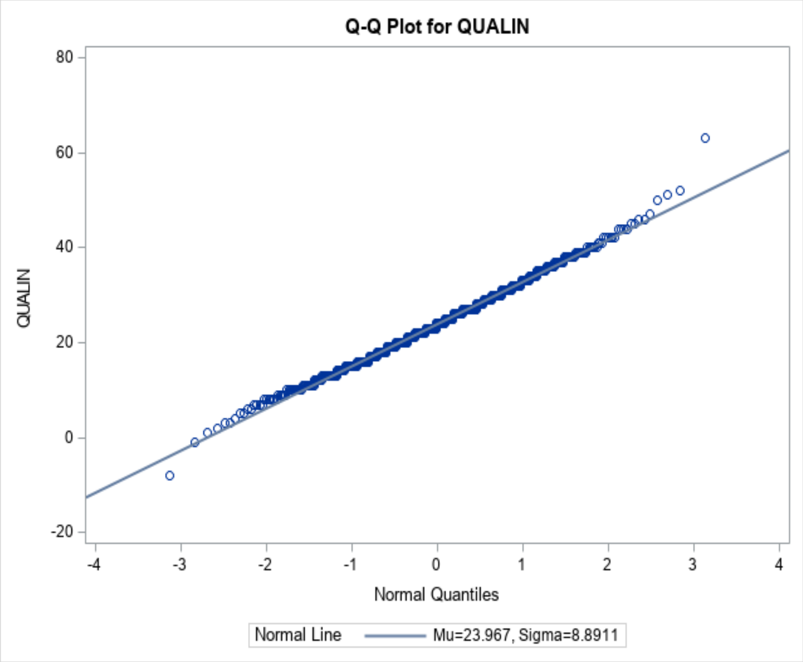

Figure S3. Q-Q Plot for QUALIN.

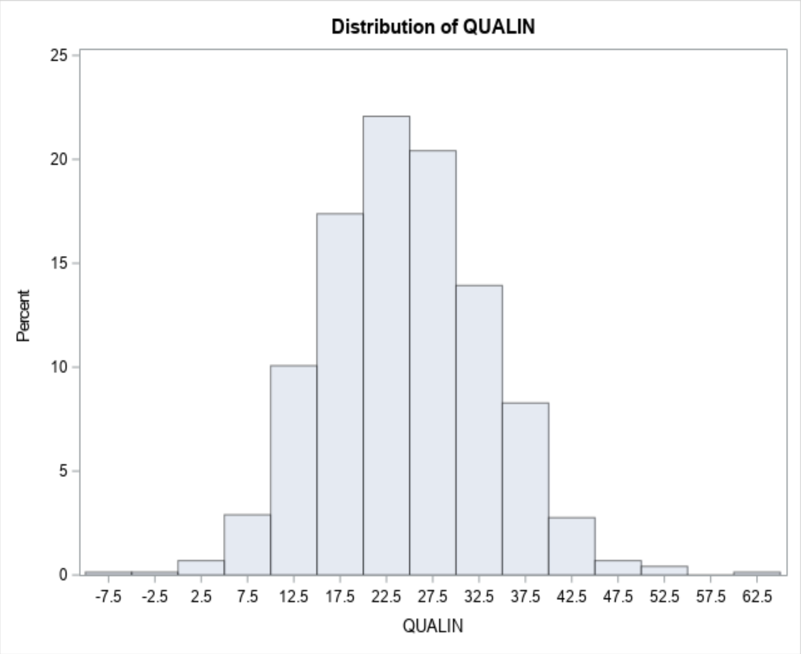

Figure S4. Distribution of QUALIN scores.
